# Residential mobility during pregnancy in the north of England

**DOI:** 10.1186/1471-2393-9-52

**Published:** 2009-11-15

**Authors:** Susan Hodgson, Mark Shirley, Mary Bythell, Judith Rankin

**Affiliations:** 1 Institute of Health and Society, Newcastle University, Newcastle upon Tyne, UK; 2 Institute of Research on the Environment and Sustainability, Newcastle University, Newcastle upon Tyne, UK; 3 Regional Maternity Survey Office, Newcastle upon Tyne, UK

## Abstract

**Background:**

Many epidemiological studies assign exposure to an individual's residence at a single time point, such as birth or death. This approach makes no allowance for migration and may result in exposure error, leading to reduced study power and biased risk estimates. Pregnancy outcomes are less susceptible to this bias, however data from North American populations indicate that pregnant women are a highly mobile group. We assessed mobility in pregnant women in the north of England using data from the Northern Congenital Abnormality Survey (NorCAS).

**Methods:**

Data were extracted from NorCAS for 1985 to 2003. Eligible cases had a gestational age at delivery of ≥ 24 weeks (a viable delivery) (n = 11 559). We assessed mobility between booking appointment (average gestational age 13 weeks) and delivery for pregnancies where the address at booking appointment and delivery were known. The impacts on mobility of maternal age and area-level socio-economic indicators were explored using standard descriptive statistics. A sensitivity analysis and a small validation exercise were undertaken to assess the impact of missing data on the estimate of mobility.

**Results:**

Out of 7 919 eligible cases for whom addresses at booking and delivery were known, 705 (8.9% (95% CI 8.3 - 9.5)) moved between booking and delivery; the mean and median moving distance was 9.7 and 1.4 km respectively. Movers were significantly younger (25.4 versus 27.3 years, p < 0.01) and lived in more deprived areas (index of multiple deprivation score 38.3 versus 33.7, p < 0.01) than non movers.

**Conclusion:**

Mobility in the north of England (9%) is considerably lower than that reported in North America and the only other study from the UK (23%). Consistent with other studies, mobility was related to maternal age and socio-economic status, and the majority of moves were over a relatively short distance. Although this population appears relatively stable, the mobility we have observed may still introduce misclassification or error into an exposure assessment relying solely on postcode at delivery, and migration should still therefore be considered a potential source of bias in future studies.

## Background

Populations are not static in space and time. People migrate into and out of a defined population or may change residential location within the population. Further, people spend daily, periodic, seasonal or longer spells away from a residence to which they later return. These movements will determine the environments people encounter as they go about their lives, and will therefore influence exposure to many environmental risk factors which vary spatially and temporally [1]. In this paper we investigate the mobility of pregnant women in the north of England using data from the Northern Congenital Abnormality Survey (NorCAS).

The importance of mobility in determining individual environmental exposures and in shaping the profile of the underlying population at risk has been acknowledged in many studies [2-7]. Nonetheless, epidemiological studies carried out at the ecological level, or using routinely collected health data, often struggle to account for the impact of mobility on exposure. When using routinely collected data, details may be available to define an individual's residence at a single time point, such as birth, hospitalisation or death. Many such studies assign exposure to an individual's residence at this time point, based on the underlying assumption that current residence in an area can be equated with exposure conditions that pertain there [3]. Such an approach makes no allowance for individuals who have migrated into (or out of) the population or for daily or periodic spells away from the current residence where different levels of exposure may be experienced. The likely result of this type of exposure assignment will be exposure error or misclassification, which will reduce the study power and may result in biased risk estimates [8].

Congenital anomalies are a diverse range of specific conditions with a spectrum of severity and occur in between 2-4% of births [9]. They are a leading cause of stillbirth and infant mortality and also a significant contributor to morbidity in childhood and beyond. Congenital anomalies have different aetiologies that are not well understood but are likely to be multifactorial, involving both genetic and environmental factors. An environmental factor may act as a mutagen in the pre-conception period, or as a teratogen during pregnancy [9]. Where environmental factors have been implicated as teratogens there is often a rather specific time frame within which exposure elicits a specific adverse effect, and for many known environmental risk factors this is often at an early stage of pregnancy when organogenesis occurs [9,10]. This relatively short period between exposure and manifestation makes congenital anomalies an appealing endpoint when assessing the effects of the environment on health.

Exposure misclassification due to migration is perceived to be most problematic for diseases with a long lag between exposure and disease onset [3,5,6]. Studies on congenital anomalies may therefore be less prone to this bias, as there is less time in which the population can migrate. However, several studies on populations in North America have indicated that pregnant women are a highly mobile group. Using data recorded in the California Birth Defects Monitoring Programme (1981-1983), Shaw and Malcoe,1992, showed that 24.8% of the women moved residence between conception and delivery [11]. Khoury et al, 1988, found that 20% of mothers moved between conception and delivery using data from the Maryland Birth Defects Reporting and Information System in 1984 [12]. Of 71 pregnant women attending public health clinics in Colorado (1996 - 1997) residential moves were made by 32% of the women [13]. A study from the USA National Birth Defects Prevention Study (1997-2000) suggested that just over 30% of expectant mothers moved house [4]. In Nova Scotia and Eastern Ontario, 12% of subjects moved between conception and delivery during 1999-2001 [14], suggesting a much lower mobility in this region. From these studies it is clear that if the postcode of the mother at the time of delivery is used as a proxy for her place of residence during pregnancy, significant exposure error will be introduced by migration if the exposure of interest impacts on the developing fetus at an early stage in the pregnancy.

The only study to look at mobility during pregnancy in the UK used data from the Office for National Statistics (ONS) Longitudinal Study comprising women (n = 6,707) who gave birth in the year immediately following the 1991 Census [15]. This study found that 23.1% of mothers moved house during pregnancy. While these findings are very interesting they may not be generalisable to the population in the north of England which, anecdotally, is considered very stable [16,17].

## Methods

The NorCAS is a prospective, population-based registry covering the former northern health region, which includes the northern counties of eastern England and northern Cumbria [see Additional file 1], and for this paper is referred to as the north of England. This region comprises a population of about three million, with approximately 35 000 births each year during the study period, of which approximately 780 births each year (2.2%) included a congenital anomaly and were therefore recorded in NorCAS [18,19]. Data are collected from this population on congenital anomalies occurring in late miscarriages at 20 weeks or more gestation, in live births and stillbirths, and in terminations of pregnancy for fetal anomaly after prenatal diagnosis, whatever the gestation at diagnosis [19,20]. Cases are reported to the register from multiple sources including antenatal ultrasound, fetal medicine, cytogenetic laboratories, the regional cardiology centre, pathology departments and paediatric surgery to ensure a high case ascertainment. Details concerning the method for data collection have been described previously [19,20]. For this study, data on all pregnancies with a congenital anomaly delivered between 1^st ^Jan. 1985 and 31^st ^Dec. 2003 were extracted from NorCAS.

The NorCAS is one of only a handful of routinely collected health datasets to hold information on residential location of each individual at more than one point in time. Details of maternal address at booking appointment (the first official check-up in pregnancy, typically at a gestational age of 13 weeks) and delivery are recorded, providing an opportunity to explore residential mobility. We acknowledge that the NorCAS data represent a specific subset of pregnancies, that this dataset may not therefore be wholly representative of all pregnancies occurring in the region, and that migration may differ between pregnancies resulting in an infant with a congenital anomaly versus those resulting in a healthy infant (see discussion).

Eligible cases were those with a gestational age at delivery of ≥ 24 weeks (a viable delivery) [21]. This cut-off was chosen as 1) it excludes most terminations and more severe (spontaneously aborted) anomalies, and 2) a bimodal plot of gestational age at delivery is more typical of congenital anomaly pregnancies. The aim was to make the findings more easily generalised to the majority of pregnancies in the region that result in a healthy baby. Where a pregnancy resulted in more than one case being entered into NorCAS (i.e. multiple pregnancies with more than one baby with anomalies), the pregnancy was counted only once (46 pregnancies affected).

The addresses at booking and delivery were geocoded based on the address postcode, a unit of postal geography comprising approximately 15 households [22]. Grid references were assigned to the postcode centroid, which represents the geographic centre of the collection of adjacent addresses making up the postcode. Grid references were obtained from the ONS All Fields Postcode Directory [23], with postcode grid references at a 100 metre resolution for the years 1985-1997, and a 1 meter resolution for the years 1998-2003. The first line of each woman's address (house number/name and street) was also compared to enable the detection of a very local move (i.e. within the same postcode), although in these cases it was not possible to calculate the distance moved.

Data on maternal age (available for 97.5% of eligible cases) was also used in this analysis. As individual level data on socio-economic status were not collected, area level census-derived socio-economic indicators (the Index of Multiple Deprivation score 2004 (IMD) [24] and Townsend deprivation score (TDS) [25]) were assigned to each mother at booking and delivery. A TDS (based on unemployment, car ownership, owner occupation, and overcrowding) calculated at the electoral ward level (a unit of administrative geography containing on average 5 500 people [22]) was assigned to each postcode based on the electoral ward the postcode fell within, and using data from the relevant Census (i.e. 1981 Census data for 1985; 1991 Census data for the years 1986-1995; 2001 Census for the years 1996-2003). An IMD score (based on crime, education, skills and training, employment, health and disability, housing and services, income, and living environment) calculated at the lower layer super output area (SOA) level (a census based geography containing on average 1 500 people [22]) was assigned to each postcode based on the SOA the postcode fell within using data from the 2001 Census.

All statistical analyses were performed using the statistical software package SPSS 15.0 (SPSS Inc., Chicago). The Chi-square test was used to compare differences in proportions, independent sample t-tests to compare means, and Pearson correlation coefficients to explore correlations between variables. Multivariable logistic regression was used to explore determinants of mobility using 'residential move' as the binary outcome; variables were entered into the model simultaneously. For the categorical variables maternal age and IMD score, where a linear dose-response relationship seemed apparent by quintiles, these variables were re-entered into the model as continuous variables to maximize power in assessing the relationship.

In almost a third of cases the address at delivery was missing, mostly over the period 1987 to 1998 (47% missing). This missing data could introduce bias into our estimates of mobility, and if the address at delivery is more likely to be missing in those cases making a residential move (i.e. is differential with respect to mobility) this could result in a substantial underestimation of mobility in this population. In addition, over the period 1999-2003, we were not able to differentiate between a woman having the same address at booking and delivery and a woman having an unknown address at delivery because of a change in data entry method (the address at booking was automatically copied to the address at delivery field, and updated only if a move was recorded to have taken place). Again, this may result in an over-estimate of the number of non movers for these years. A sensitivity analysis was undertaken to assess the impact on mobility of applying different assumptions to these apparent non movers over the period 1999-2003, and to the missing data over the period 1987-1998. In addition, a small validation exercise was undertaken by cross-referencing a sample of randomly selected cases with the UK National Health Service National Strategic Tracing Service (NSTS) records. From this validation it was possible to assess whether moves had taken place during pregnancy when the NorCAS addresses at booking and delivery were the same, either because no move had taken place, or because they were automatically copied across and would previously have been unknown.

### Ethical approval

The NorCAS has Patient Information Advisory Group exemption from a requirement for consent for inclusion on the register under section 60 of the Health and Social Care Act (2001) and has ethics approval (04/MRE04/25) to undertake studies involving the use of its data.

## Results

Out of 14 885 cases in the NorCAS dataset over the period 1985-2003, 11 559 (77.7%) referred to unique pregnancies with a gestational age at delivery of ≥ 24 weeks, and were therefore eligible for analysis. For these eligible pregnancies the mean gestational age at booking, recorded from 1999 onwards, was 12.7 weeks (Standard Deviation (SD) 5.58). The mean gestational age at delivery, available from 1985 onwards, was 37.9 weeks (SD 3.37), and the mean number of weeks between booking and delivery over the period 1999-2003 was 23.6 (SD 6.81). The mean maternal age at delivery was 27.1 years (SD 5.89), and there was a clear trend of increasing maternal age over the study period from 25.9 (SD 5.3) years in 1985 to 27.9 (SD 6.3) years in 2003 (p < 0.01) (data not shown).

Of these eligible pregnancies, the address at booking and delivery were known for 7 919 (68.5%). The mean maternal age at delivery was slightly higher (p = 0.01) and the mean gestational age at delivery slightly lower (p < 0.01) in those pregnancies for whom the address at booking and delivery were known compared to those in which they were unknown. The area-level measures of socio-economic status did not differ significantly between these groups (table 1).

**Table 1:** Characteristics of all eligible pregnancies; pregnancies where address at booking and delivery known (included in analyses as non movers and movers); and pregnancies where address at booking and/or delivery not known (not included in the analyses).

Variable	All eligible pregnancies		Pregnancies where address at booking and delivery known (n = 7 919)						Address at booking or delivery not known	
	(n = 11 559)		Non movers and movers (n = 7 919)		Non movers (n = 7 214)		Movers (n = 705)		(n = 3 640)	
	**N**	**Mean (SD)**	**N**	**Mean (SD)**	**N**	**Mean (SD)**	**N**	**Mean (SD)**	**N**	**Mean (SD)**

Maternal age at delivery	11269	27.1 (5.9)	7746	27.2 (6.0)	7048	27.3 (6.0)	698	25.4 (6.0)	3523	26.8 (5.6)

Gestational age at delivery	11559	37.9 (3.4)	7919	37.8 (3.4)	7214	37.8 (3.5)	705	37.6 (3.4)	3640	38.1 (3.2)

IMD* at booking	11449	33.9 (18.4)	7878	34.1 (18.3)	7178	33.7 (18.2)	700	38.3 (18.4)	3571	33.5 (18.5)

TDS** at booking	11451	3.2 (3.9)	7857	3.2 (3.9)	7160	3.2 (3.9)	697	3.9 (4.0)	3594	3.1 (3.9)

* IMD = Index of multiple deprivation

** TDS = Townsend deprivation score

Of the eligible women for whom the address at booking and delivery were known, 705 (8.9% (95% Confidence Interval (CI) 8.3 - 9.5)) moved between booking and delivery. Of the women who moved, addresses at booking and delivery were geocoded for 672 (95.3%), revealing a mean and median moving distance of 9.7 and 1.4 km respectively; the majority of moves were made within a relatively short distance (table 2) [and see Additional file 2].

**Table 2:** Residential migration in the NorCAS* dataset 1985-2003.

Residential migration	Frequency	%	95% Confidence Interval
No move	7214	91.10	90.5 - 91.7

Moved	705	8.90	8.3 - 9.5

Of the women who moved:			

Moved within postcode	29	4.11	2.6 - 5.6

Moved up to 500 m	154	21.84	18.8 - 24.9

Moved 500- < 1000 m	99	14.04	11.5 - 16.6

Moved 1- < 2 km	107	15.18	12.5 - 17.8

Moved 2- < 5 km	129	18.30	15.4 - 21.2

Moved 5- < 10 km	75	10.64	8.4 - 12.9

Moved ≥ 10 km	79	11.21	8.9 - 13.5

Unable to geocode	33	4.68	3.1 - 6.2

Total	705	100	

*NorCAS = Northern Congenital Abnormality Survey

The mean maternal age at delivery was significantly lower in women who moved compared to women who did not move (p < 0.01) (table 1). When mobility was assessed by age group, the percentage of women moving house decreased with increasing age group (p < 0.01), but increased again slightly in the oldest age group (≥ 35 years); see table 3.

**Table 3:** Multivariable logistic regression analysis showing variables influencing the likelihood of residential moves between booking and delivery.

Variable		n	OR	95% CI		p
				**Lower**	**Upper**	

Delivery year		7919	1.03	1.01	1.04	< 0.01

Maternal age at delivery (continuous variable)		7746	0.95	0.94	0.96	< 0.01

Maternal age at delivery	< 20	859	2.01	1.47	2.77	< 0.01

	20 - 24	1888	1.66	1.25	2.22	< 0.01

	25 - 29	2334	1.11	0.83	1.48	0.48

	30 - 34	1723	0.68	0.49	0.94	0.02

	> = 35 (reference)	942	1.00	-	-	-

IMD* score at booking (continuous variable)		7878	1.01	1.00	1.01	< 0.01

IMD* score at booking (quintiles)	1 (most affluent (reference))	1475	1.00	-	-	-

	2	1555	1.02	0.76	1.36	0.94

	3	1621	1.13	0.85	1.49	0.40

	4	1594	1.33	1.01	1.75	0.04

	5 (most deprived)	1633	1.52	1.16	1.99	< 0.01

* IMD = Index of multiple deprivation

Women who moved house between booking and delivery had a higher average IMD score (i.e. were more deprived) than women who did not move (p < 0.01) (table 1). The percentage of women moving house increased linearly with increasing quintile of IMD score at booking (p < 0.01); see table 3 [and see Additional file 3].

In those women that moved, there was a strong correlation between IMD score at booking and delivery (Pearson correlation coefficient = 0.54, p < 0.01), however the IMD score at delivery was marginally, but significantly, lower than at booking (37.4 versus 38.8 at delivery and booking respectively, p < 0.05).

The IMD score at booking was negatively associated with maternal age (Pearson correlation coefficient = -0.273, p < 0.01), and IMD score decreased with increasing maternal age group (p < 0.01) (data not shown). Very similar trends were obtained when TDS was used instead of IMD score as the measure of socio-economic deprivation.

Multivariable logistic regression indicated that increasing year of delivery increased the likelihood of moving house, whilst increasing maternal age decreased the likelihood of moving house. Higher IMD scores at booking were associated with an increased likelihood of a residential move (see table 3).

**Figure 1:**
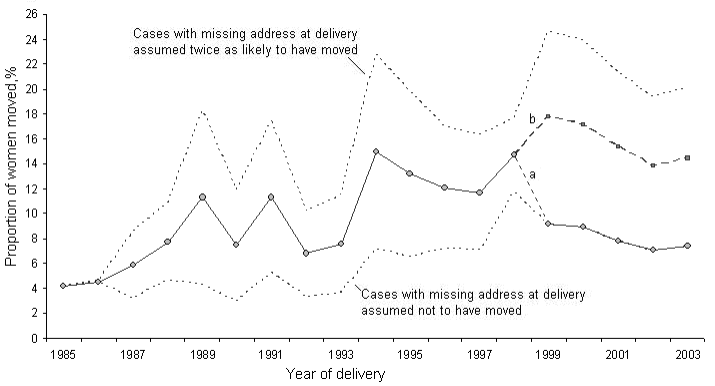
**Percentage of women moving house during pregnancy by year of delivery**. The solid line up to 1999 shows mobility when cases with a missing address at delivery are excluded from the analysis; the dotted lines show mobility under the assumption that cases with missing address at delivery had not moved (lower line), or that cases with missing address at delivery were twice as likely to have moved (upper line). From 1999, due to changes in data entry, cases with the same address at booking and delivery were a) assumed not to have moved or b) re-classified to represent missing data (47%) and excluded from the analysis.

A sensitivity analysis was undertaken to assess the impact on mobility of applying different assumptions to the apparent non movers over the period 1999-2003, and to the missing data over the period 1987-1998 (figure 1). For the period 1999-2003 we assumed that 47% of the cases where no move was recorded actually refer to cases where the address at delivery was not known (and which would, as in previous years, have been excluded from the analysis). Under this scenario the estimate of mobility increases from 8.9 to 10.8% (figure 1, scenario b). For the missing data over the period 1987-1998 two assumptions were made. Firstly, all cases with a missing address at delivery were assumed not to have moved (i.e. assumed to have the same address at delivery as at booking). Secondly, all cases with a missing address at delivery were assumed to have been twice as likely to have moved, to assess the possible bias that might have been introduced if the women with missing addresses were more likely to have moved out to the area. Under the first assumption, the overall percentage of women migrating during pregnancy was calculated to be 6.1%; under the second assumption the percentage of movers was calculated to be 15.3%; the best estimate of mobility in this dataset is therefore likely to be somewhere between these two figures.

A small sample, n = 50 randomly selected cases, were cross-referenced with the NSTS records. Out of these 50 cases, 5 were found to have moved during pregnancy; 3 at gestational ages likely to fall between booking and delivery (at weeks, 9, 13 and 35), and 2 very early in pregnancy (2 and 5 weeks), probably before booking.

## Discussion

We have shown that the residential mobility of pregnant women in the north of England is considerably lower than that reported in North America [4,11-14], and lower than the only previous figure of 23.1% quoted for the UK [15]. Consistent with these other studies, we found that mobility was higher in younger women [4,12,14] and in women living in more deprived areas [4,14] and that the majority of moves were over a relatively short distance [11,13-15]. In addition, we found that, overall, residential moves were made to less deprived areas. These data support the anecdotal evidence that this population is comparatively stable. We have used prospectively collected data from a long-standing, high quality register of congenital anomalies that uses multiple sources for case ascertainment. However, there are several limitations of these data which may restrict the generalisability of our results.

The NorCAS data represent a specific subset of pregnancies, namely those affected by a congenital anomaly, and as such this dataset may not be wholly representative of all pregnancies occurring in the region, most of which result in a healthy infant. For pregnancies where a congenital anomaly is first identified at birth, it seems unlikely that migration patterns would differ from those exhibited by any other pregnant woman. However it is possible that the likelihood of migration could be affected if the health status of the unborn infant is known prior to birth. In such instances we might observe a higher rate of 'migration for care', perhaps to areas with specific health care facilities, or to be nearer family members [3]. Despite this possible bias, several studies have shown that the patterns of migration during pregnancy are similar in mothers of infants with and without congenital anomalies [4,11], suggesting that the mobility observed in the NorCAS dataset should be generalisable to all pregnant women in the region.

To assess mobility during pregnancy and to draw appropriate comparisons with findings from other studies, we would need to investigate maternal moves made between conception and delivery. As the NorCAS data does not currently include details of address at conception, this assessment is not possible. Our findings relate to mobility between booking and delivery, which reflects a period of 24 weeks on average, or about 60% of the pregnancy, and maternal moves taking place prior to booking have not been assessed. The percentage of women moving house in the period between booking and delivery is likely to be lower than the percentage of moves taking place throughout the whole pregnancy, and so the finding of 8.9% of women moving house may be an underestimate of mobility throughout pregnancy. The study by Fell et al (2004) reported a mobility of 12% throughout the entire pregnancy, but only 3% in the first trimester, and 9% through the second and third trimesters combined [14]. If similar trends can be assumed to apply to the NorCAS women, we should have captured the majority of moves taking place. Dolk et al (1997) also provide data on maternal moves at various stages in pregnancy showing that 23.1% of women moved throughout pregnancy, and that 19.2% moved in the last six months of pregnancy [15]. This latter figure reflects a similar period to that captured between booking and delivery in the NorCAS data extract, and further supports our view that even if the figure of 8.9% is an underestimate, the mobility of pregnant women in the north of England is still relatively low compared to women throughout the UK.

As almost a third of cases were missing an address at delivery our assessment of mobility could be biased if these missing data are related to migration out of the region. We carried out sensitivity analyses to assess the impact of various scenarios on our overall estimate of migration. Based on this assessment it seems probable that a lower realistic figure of mobility in this dataset is 6.1%, and a higher realistic figure is 15.3%; the best estimate of mobility in this dataset is likely to be somewhere between these two figures, most likely between 8.9 and 10.9%. The small validation exercise suggests that over the period 1999-2003 approximately 6% of apparent non movers did actually move between booking and delivery, which would revise the best estimate of mobility to approximately 10.9%.

## Conclusion

While our findings support anecdotal evidence that this is a stable population, this relatively low mobility may still introduce misclassification or error into an exposure assessment relying solely on postcode at delivery. Migration should still therefore be considered a potential source of bias in studies reliant on a single residential identifier relating to location at the end of pregnancy, to classify exposure. Given the local nature of most house moves made by pregnant women, this bias is likely to be minimal when calculating regional or district-level rates of congenital anomalies. However, when assessing the impact of a local point source of pollution on congenital anomaly risk, even local moves may introduce exposure error and result in biased risk estimates.

As data are often unavailable on residences throughout pregnancy for populations across the UK, and in many other countries, it is impossible to accurately assess mobility during pregnancy, or to explore the impact of the bias introduced into epidemiological studies by this migration. In populations for which valuable residential history data are available, such as that covered by the NorCAS, the impact of this bias can be explored and insights gained applied to other populations.

## Competing interests

The authors declare that they have no competing interests.

## Authors' contributions

SH designed and coordinated the study, carried out the data analyses and interpretation, and drafted the manuscript. MS was responsible for data cleaning and linkage of the NorCAS cases to the socio-economic data. MB was responsible for the NorCAS database and cross-referencing the NorCAS data with the National Strategic Tracing Service records. JR conceived the study, participated in its design and coordination and helped to draft the manuscript. All authors read and approved the final manuscript.

## Pre-publication history

The pre-publication history for this paper can be accessed here:

http://www.biomedcentral.com/1471-2393/9/52/prepub

## Supplementary Material

Additional file 1 **Map showing the geographic coverage of the Northern Congenital Abnormality Survey (NorCAS) (shaded area)**. Click here for file

Additional file 2 **Scatter diagram of residential moves made between booking and delivery**. In this figure 93% of the movers represented, the remaining 7% moved further afield. Each point represents one woman making a residential move away from her address at booking (addresses at booking have been set to the centre point of the plot). Scatter diagram showing residential moves made between booking and delivery. Click here for file

Additional file 3 **Percentage of women moving between booking and delivery by quintile of IMD score at booking**. Box plot showing percentage of women moving between booking and delivery by quintile of IMD score at booking. Click here for file
