# Supplementary material for: Residential mobility during pregnancy in the north of England
Source: BMC Pregnancy Childbirth. 2009 Nov 15;9:52. doi: 10.1186/1471-2393-9-52 (PMC2784435; doi:10.1186/1471-2393-9-52)

Additional file 1: Map showing the geographic coverage of the Northern Congenital Abnormality Survey (NorCAS) (shaded area).


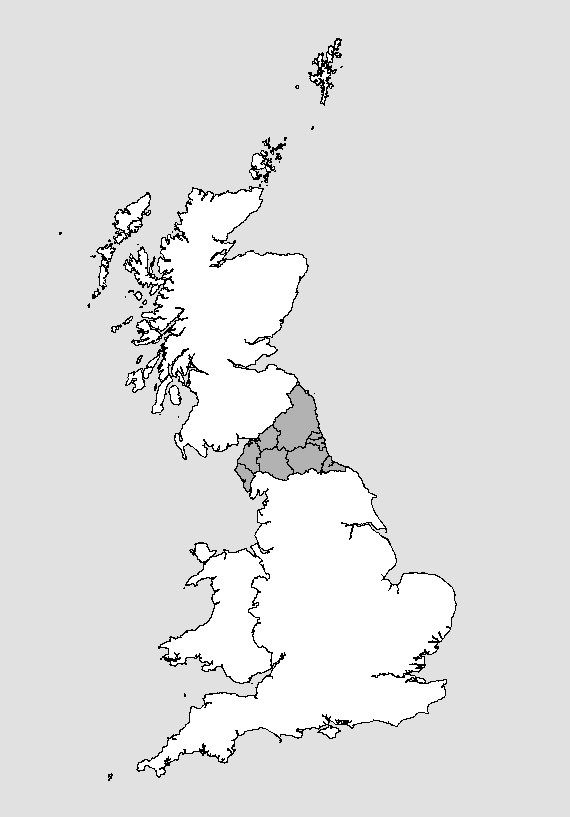

Supplement: Additional file 1 — Map showing the geographic coverage of the Northern Congenital Abnormality Survey (NorCAS) (shaded area). [file 1471-2393-9-52-S1.DOC]
