# Supplementary material for: Residential mobility during pregnancy in the north of England
Source: BMC Pregnancy Childbirth. 2009 Nov 15;9:52. doi: 10.1186/1471-2393-9-52 (PMC2784435; doi:10.1186/1471-2393-9-52)

Additional file 2: Scatter diagram of residential moves made between booking and delivery.

In this figure 93% of the movers represented, the remaining 7% moved further afield. Each point represents one woman making a residential move away from her address at booking (addresses at booking have been set to the centre point of the plot).


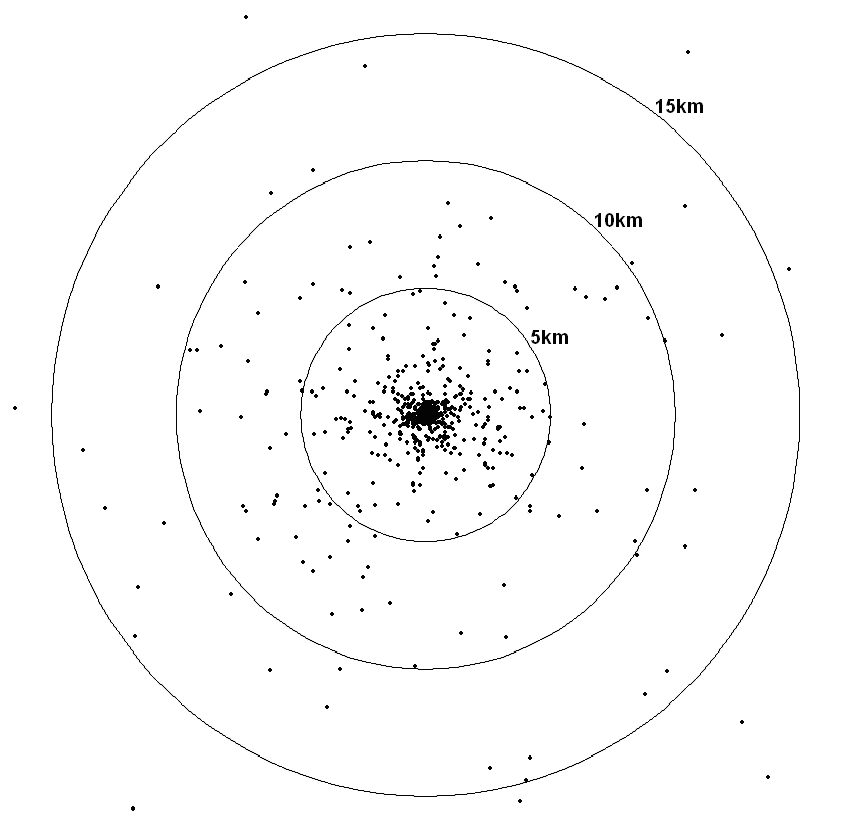

Supplement: Additional file 2 — Scatter diagram of residential moves made between booking and delivery. In this figure 93% of the movers represented, the remaining 7% moved further afield. Each point represents one woman making a residential move away from her address at booking (addresses at booking have been set to the centre point of the plot). Scatter diagram showing residential moves made between booking and delivery. [file 1471-2393-9-52-S2.DOC]
