# Supplementary material for: Residential mobility during pregnancy in the north of England
Source: BMC Pregnancy Childbirth. 2009 Nov 15;9:52. doi: 10.1186/1471-2393-9-52 (PMC2784435; doi:10.1186/1471-2393-9-52)

Additional file 3: Percentage of women moving between booking and delivery by quintile of IMD score at booking.


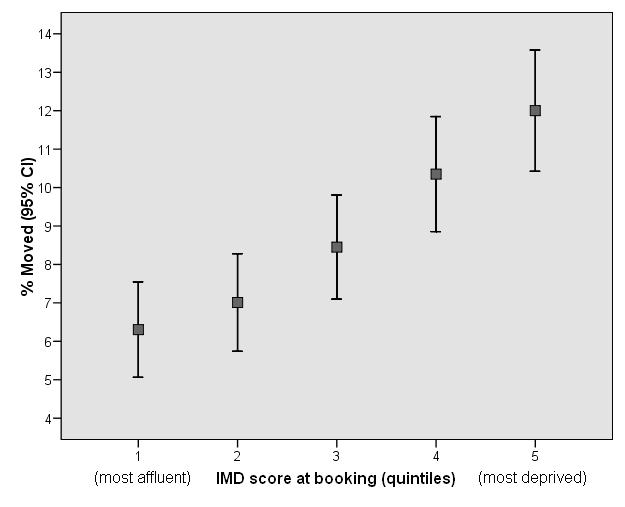

Supplement: Additional file 3 — Percentage of women moving between booking and delivery by quintile of IMD score at booking. Box plot showing percentage of women moving between booking and delivery by quintile of IMD score at booking. [file 1471-2393-9-52-S3.DOC]
